# Marine Bioactives: Pharmacological Properties and Potential Applications against Inflammatory Diseases

**DOI:** 10.3390/md10040812

**Published:** 2012-04-05

**Authors:** Nicolantonio D’Orazio, Maria Alessandra Gammone, Eugenio Gemello, Massimo De Girolamo, Salvatore Cusenza, Graziano Riccioni

**Affiliations:** 1 Human Nutrition, Department of Biomedical Science, via Dei Vestini, University G. D’Annunzio, Chieti, 66013, Italy; 2 Cardiology Unit, San Camillo De Lellis Hospital, Manfredonia, FG, Italy

**Keywords:** inflammation, inflammatory diseases, marine bioactives, oxidative stress, reactive oxygen species, marine carotenoids, antioxidants

## Abstract

Inflammation is a hot topic in medical research, because it plays a key role in inflammatory diseases: rheumatoid arthritis (RA) and other forms of arthritis, diabetes, heart diseases, irritable bowel syndrome, Alzheimer’s disease, Parkinson’s disease, allergies, asthma, even cancer and many others. Over the past few decades, it was realized that the process of inflammation is virtually the same in different disorders, and a better understanding of inflammation may lead to better treatments for numerous diseases. Inflammation is the activation of the immune system in response to infection, irritation, or injury, with an influx of white blood cells, redness, heat, swelling, pain, and dysfunction of the organs involved. Although the pathophysiological basis of these conditions is not yet fully understood, reactive oxygen species (ROS) have often been implicated in their pathogenesis. In fact, in inflammatory diseases the antioxidant defense system is compromised, as evidenced by increased markers of oxidative stress, and decreased levels of protective antioxidant enzymes in patients with rheumatoid arthritis (RA). An enriched diet containing antioxidants, such as vitamin E, vitamin C, β-carotene and phenolic substances, has been suggested to improve symptoms by reducing disease-related oxidative stress. In this respect, the marine world represents a largely untapped reserve of bioactive ingredients, and considerable potential exists for exploitation of these bioactives as functional food ingredients. Substances such as *n*-3 oils, carotenoids, vitamins, minerals and peptides provide a myriad of health benefits, including reduction of cardiovascular diseases, anticarcinogenic and anti-inflammatory activities. New marine bioactives are recently gaining attention, since they could be helpful in combating chronic inflammatory degenerative conditions. The aim of this review is to examine the published studies concerning the potential pharmacological properties and application of many marine bioactives against inflammatory diseases.

## 1. Introduction

Inflammation has different names in different parts of the body: rhinitis (inflammation of the nose), asthma (inflammation of the airways), arthritis (inflammation of the joints), dermatitis (inflammation of the skin), and so on. As the initial response that fires up the immune system, inflammation is the crucial first step in fighting infection and healing wounds. However, persistent inflammation and an immune system that is always activated, is known as chronic inflammation, leading to chronic disease. Inflammation also plays a role in heart disease [1], because the immune system attacks the “bad” cholesterol (LDL) incorporated in arterial walls. Ongoing inflammation eventually damages the arteries: it is so closely associated with heart disease that a test for inflammation called CRP (C-reactive protein) is used to assess cardiovascular risk, predicting the risk of Coronary Heart Disease (CHD) and stroke, together with cholesterol levels [2]. 

For example, RA, a chronic progressive autoimmune disease, characterized by erosive painful symmetric synovitis with possible multisystem involvement, involves inflammation. In fact, some highly reactive transient chemical species, called reactive oxygen species (ROS), are involved in its pathogenesis, and pro-inflammatory cytokines, such as TNF-α and IL-1β, are implicated in the formation of toxic peroxynitrite by increasing the activity of nitric oxide synthase (NOS). The ROS trigger a cascade of events through NF-κB activation, which up-regulate gene expression of proinflammatory cytokines that activates immune responses, determining inflammation and cartilage damage [3]. 

Inflammation has also been linked to diabetes. In type 1 diabetes, the immune system attacks the cells that make insulin; type II diabetes is also linked to inflammation, as chronic inflammation induces the release of TNF- α, which makes cells more resistant to insulin [4]. 

In addition, even a link between inflammation and cancer exists: protein p100 allows communication between the inflammation and development processes, but in the case of chronic inflammation, the presence of too much p100 over-activates the developmental pathway, resulting in cancer [5].

Considering the involvement of phlogistics mediators, many inflammatory diseases could potentially be alleviated by dietary modification; diet may represent a potential therapeutic agent. 

Many reviews suggested that there is evidence of a protective effect of higher consumption of olive oil, fruits and vegetables, for example in developing RA, since dietary antioxidants effectively suppress the release of inflammatory cytokines by reducing ROS production [6]. The role of food in improving health has been recognized, activating the development of new classes of food, known as functional foods [7], which could decrease the risk of illness and the incidence or severity of chronic inflammatory disorders [8,9]. Numerous marine bioactives have been recently identified, whose several biological activities could interfere with the pathogenesis of many diseases. It has already been shown that bioactive peptides isolated from fish protein hydrolysates, algal fucans, galactans and alginates possess anticoagulant, anticancer and hypocholesterolemic activities [10]. Fish oils and marine bacteria are known to be excellent sources of omega-3 fatty acids (whose importance in the treatment of arthritis has been extensively investigated [11,12], assessing their analgesic effects in joint pain), while seaweeds and crustaceans contain powerful antioxidants, such as carotenoids and phenolic compounds [13]. In this respect, new marine bioactives, such as COX inhibitors (Pacifenol, Epitaondiol and Stypotriol triacetate), marine steroids (Contignasterol, Xestobergesterol, Clathriol B), molecules interfering with NF-κB (Cycloprodigiosine, Hymenialdisine and Cycloamphilectenes), macrolides, peptides (Cyclomarins, Salinamides and Halipeptins), other metabolites (Scytonemin and Petrocortyne) and many antioxidant agents (phenols and marine carotenoids, such as astaxanthin, fucoxanthin) have been recently discovered and characterized, in order to assess their potential role in contrasting inflammatory diseases. 

## 2. Role of Oxidative Stress and Antioxidants in Inflammatory Diseases

There is a close relationship between oxidative stress (OS) and inflammation in patients with inflammatory diseases. In most phlogistic conditions, macrophages and leukocytes are activated firstly, so that ROS are generated in excessive amounts, determining OS. Elevated level of ROS, such as superoxide anion, nitric oxide (NO), hydrogen peroxide and hydroxyl radical, in synovial fluid (generated by activated macrophages, monocytes, and granulocytes, as well as anoxic reperfusion reactions), plasma malondialdehyde (MDA) and degradation products of lipid peroxidation represent important characterizing factors of disease [6].

This is also demonstrated by increased plasma malondialdehyde (MDA) levels observed in previously published reports as end products and an indirect indicator of increased ROS generation [14]. Furthermore, during chronic inflammation, even protective mechanisms increase to dangerous levels: higher concentrations of ROS cause substantial lipid peroxidation, leading to toxic tissue damage [15]. In addition, some antioxidant erythrocyte enzymes, such as glutathione peroxidase (GSH-Px), superoxide dismutase (SOD) and catalase (CAT) in RA patients have been shown to have lower activities than in healthy subjects [16]. 

Previous investigations have also established a relationship between systemic markers of inflammation and serum beta-carotene levels, and that phlogistic conditions produce increased ROS and decreased antioxidant levels, which may worsen the symptoms of disorders such as RA, osteoarthritis and systemic lupus erythematosus [17,18]; some authors implied a deficient level of vitamin E among causes of their development. Decreased serum concentrations of α-tocopherol, and β-carotene were suggested as possible risk factors [19]. To confirm this, vitamin E supplementation was shown to improve clinical symptoms of RA, probably by reducing the formation of prostaglandins during the phlogistic process [20]. In fact, the combination of standard RA treatment and antioxidants even increases serum GSH-Px activity with subsequent clinical improvement, including in joint pain and morning stiffness [21]. 

Based on these reports, improvement in antioxidant status through a greater intake of dietary antioxidants such as vitamin E, vitamin C, carotenoids (including β-carotene, β-cryptoxanthin, and zeaxanthin) and phenols, may prevent RA development and help RA management. Circulating antioxidants are scavengers of free radicals (FRs), and may inhibit oxidative damage and lead to the abolition of inflammation [22,23]. They can not only alleviate symptoms by reducing disease-related OS, but can also ameliorate the potential side-effects and reduce the risk of complications of pharmacological therapy (non-steroidal anti-inflammatory drugs and corticosteroids) such as gastrointestinal bleeding, bone loss and increased requirement of some nutrients. It was recently postulated that circulating antioxidants also have a role in the prevention of cardiovascular disease (CVD): C-reactive protein (CRP) and oxidized LDL-cholesterol concentrations, are inversely related to plasmatic concentrations of vitamin C, carotenoids and phenols [24,25,26]. Therefore, dietary antioxidants may be protective against the development of inflammatory disease [27].

## 3. Biology of Marine Natural Products: A Potential Anti-Inflammatory and Antioxidant Strategy?

Most anti-inflammatory drugs used against inflammatory disorders are cyclooxygenase (COX) inhibitors. They usually exert analgesic effects: non-steroidal anti-inflammatory agents, such as aspirin and indomethacin, relieve pain by inhibiting the production of prostaglandins and decreasing the sensitivity of peripheral nociceptors [28]. The enzyme COX-1 is involved in the analgesic effect [29]; it produces prostaglandins, which protect the kidney and stomach from tissue and mucosal damage. Conversely, inhibition of COX-1 causes renal damage and gastric irritation, the typical side effects of aspirin-like drugs [30]. The other enzyme, COX-2, requires more time than COX-1 to form prostaglandins and contributes to the inflammation. However, among inflammation-related targets, we should consider not only COX, but also molecules able to interfere with factors involved in the modulation of gene expression, such as NF-κB, which could also act as potential anti-inflammatory agents [27]. In this respect, marine natural bioactives were recently shown to contain antioxidant agents, steroids and several novel molecular entities potentially able to target COX-1, COX-2 and the NF-κB pathway [31].

### 3.1. COX Inhibitors

#### 3.1.1. Pacifenol

Pacifenol is a terpenoid isolated from seaweeds of the marine alga *Laurencia claviformis*, collected in Easter Island and belonging to the Rhodomelaceae family, whose structure was characterized by chemistry studies and crystallographic analysis in 1971 [32]. 

The probable precursor of pacifenol, prepacifenol, was originally isolated from the Australian red alga *Laurencia filiformis*, but later, pacifenol was found naturally in *Laurencia tasmanica* as well [33]. This halogenated sesquiterpene represented the first example in chemical literature of a natural bioactive obtained from algae containing bromine and chlorine atoms covalently bound [34]. 

However, these new metabolites, pacifenol and prepacifenol, were found not only in red alga, but also in some marine invertebrates, for example in the digestive system of the mollusk *Aplysia californica* [35]. 

An antimicrobial activity of pacifenol derivatives has previously been reported, after testing against some microrganisms, especially against *Pseudomonas aeruginosa* and *Streptococcus enteriditis*.

In addition, pacifenol exerts an inhibitory activity on inflammation by decreasing leukotriene B4 (LTB4) and thromboxane B2 (TXB2) production, but also stopping degranulation response [36]. 

In particular, its anti-inflammatory action, exercised through inhibition of the key enzyme phospholipase A2 and the consequent modulation of the cyclooxigenase pathway, and its anti-allergy effect, could be exploited to contrast the phlogistic processes implicated in the pathogenesis of many inflammatory and allergic diseases.

#### 3.1.2. Epitaondiol

This terpenoid was isolated from seaweeds of *Stypopodium flabelliforme*, collected near Easter Island in the South Pacific Ocean [37]. The genus Stypopodium is a tropical group of brown algae, phaeophyceae, with rich components of polycyclic meroditerpenoids, possessing several biological activities [38]. Epitaondiol diacetate showed pharmacological effects in the rat cardiovascular system; a negative inotropic and chronotropic effect was noticed [39], but it also revealed marked anti-inflammatory effects through inhibition of eicosanoids (LTB4 and TXB2) release and modulation of the cyclooxigenase pathway. This happens through inhibition of the key enzyme phospholipase A2, which plays an important role in the release of arachidonic acid and the formation of lipid mediators [40]. Its anti-inflammatory activity is stronger than that of indomethacine [41]. 

Epitaondiol also showed dose-dependent gastroprotective activity in mice gastric lesions, displaying similar action to lansoprazole [42]. 

This double effect, both anti-inflammatory and gastroprotective, could be a fascinating treatment strategy without the well known side effects of drugs which are conventionally prescribed for the treatment of inflammatory diseases.

In addition, epitaondiol exhibited antimicrobial effects against gram-positive and gram-negative bacteria, especially against *E. faecalis *[43], antiviral activity against herpes simplex [44], and antiproliferative properties: human colorectal adenocarcinoma and neuroblastoma cell line showed higher susceptibility [45]. In particular, 2beta-3alpha-epitaondiol possesses sodium channel blocking activity, a cytotoxic active against human lung cancer cells [46]. 

Instead, the similar molecule called isoepitandiol showed a radical scavenging activity even more powerful than ascorbic acid [47]. 

Both its powerful anti-inflammatory activity and this strong antioxidant effect deserve further studies, since they may be helpful in targeting the inflammation and oxidative stress that characterize many chronic inflammatory disorders.

#### 3.1.3. Stypotriol Triacetate

This polycyclic meroditerpenoid [48], the complete configuration of which was recently determined [49], was also isolated from the seaweed *Stypopodium flabelliforme*. It is unstable in air, so that it had to be prepared by acetylation of the organic extract to avoid air oxidation in order to study its characterization and biological activities [50]. 

*Stypotriol* displayed antifeedant and anti-inflammatory activity [51]. In particular, it exerts an inhibitory activity on inflammation by interfering with elastase release, by modulating the cyclooxigenase pathway through inhibition of phospholipase A2, and by decreasing the secretion of eicosanoids [40]. This may be useful in inhibiting inflammation and reducing elastase-induced cartilage degradation and articular damage, typical of RA and responsible of pain and loss of joint function.

Similarly to epitaondiol, it showed antibacterial effects against gram-positive and gram-negative bacteria [43], and antiproliferative properties (human colorectal adenocarcinoma and rat basophilic leukemia cell line showed higher susceptibility) and was the most cytotoxic toward cancer cells with a concentration-dependent inhibitory effect, followed by epitaondiol [45].

### 3.2. Marine Steroids

Steroids are synthetic drugs widely used for treating asthma, RA, psoriasis and a wide variety of inflammatory conditions. They work by decreasing inflammation and reducing the activity of the immune system; they provide significant relief from articular pain and stiffness, dyspnea, cutaneous manifestation and other phlogosis related symptoms.

Marine organisms, in particular sponges, have recently been recognized as a notable source of uncommon steroids showing potent biological anti-inflammatory activities.

#### 3.2.1. Contignasterol

This natural polyoxygenated steroid with a new side chain, isolated from the marine sponge *Petrosia contignata* in Papua New Guinea, has been the subject of many investigations, including both biological studies and synthetic work [52].

It belongs to steroid class but it has a particular chemical structure, because of the unusual set of functional groups, the details of which have been already published [53]. Study results have shown its potential value in the treatment of asthma and other inflammatory diseases [27]. In particular, it inhibits the release of histamine from human basophils and lung tissue and attenuates the contractile response to histamine, probably indirectly interacting with cellular signaling systems leading to the inhibition of phospholipase C activity [31], protecting in this way from bronchoconstriction [50]. 

In addition, contignasterol showed an ability to inhibit platelet aggregation in response to their activating factor PAF, which is a local mediator of thrombotic events, and collagen exposure of vessels, suggesting anti-thrombolytic activity. As a consequence, the pharmacological potential of contignasterol could enable it to be used as a cardiovascular and antiallergic drug, in order to treat hemodynamic disorders involving platelets, hypertension or hypotension, thrombosis, asthma, allergic rhinitis, psoriasis, rashes, osteoarthritis and inflammation in general [54,55].

#### 3.2.2. Xestobergsterol

This pentacyclic polyhidroxylated steroid was isolated in 1992 from the Okinawan marine sponge *Xestospongia bergquisita* [56]. 

It is a strong inhibitor of IgE-mediated histamine release from activated mast cells [57], with an inhibitory effect that is much more potent than the antiallergy drug disodium cromoglicate [53]. 

In particular, Xestobergsterol A dose-dependently inhibited the generation of inositol triphosphate (IP3) and phospholipase C (PLC) activity and inhibits Ca^2+^-mobilization from intracellular Ca^2+^-stores, which are early events in IgE-dependent mediator release [58]. Xestobergsterol has undergone a number of investigations, including synthetic work on its analogues [59].

So, like contignasterol, it could be considered a potential anti-asthma agent with a promising pharmacological potential [27]. 

#### 3.2.3. Clathriols

Clathriols A and B are novel polyoxygenated steroids isolated from the marine sponge *Clathria lissosclera*, in New Zealand waters. 

They possess the rare and only naturally-occurring 14-beta-stereochemistry, a typical configuration of marine sponges [60]: This makes them structurally and biologically very similar to contignasterol, even if their biological action has turned out to be less powerful than contignasterol and its derivatives in blocking histamine release [27].

Both are not only light anti-allergy molecules, but also moderate anti-inflammatory compounds. In particular, the anti-inflammatory pharmacology of clathriol B was reported in 2000 [61]; however, further studies took place during 2003 [60] and more recently [62,63]. 

Clathriol B inhibits the production of superoxide from human peripheral blood neutrophils [50], which is known to be implicated in the pathogenesis of inflammatory disorders: in this respect also clathriols should be studied in more depth, since they may also be helpful in alleviating the inflammation and oxidative stress that characterize several chronic degenerative diseases. 

### 3.3. Molecules Interfering with NF-κB

#### 3.3.1. Cycloprodigiosin

This molecule belongs to the prodigiosin family, but here, the side chain is cyclized to form a six-membered ring [64]. Cycloprodigiosin is the red pigment produced by various marine bacteria, including *Serratia marcenses*, *Zooshikella rubidus *and *Pseudoalteromonas denitrificans*, with immunosuppressive properties and apoptotic effects on cancer cells, interacting with p65 and the nuclear factor κB (NF-κB) pathway [65]. In particular, Cycloprodigiosin hydrochloride, produced by *Pseudoalteromonas denitrificans*, causes cytotoxic effects and apoptotic cell death in various cancerous cell lines, especially with the pro-inflammatory cytokine Tumor Necrosis Factor (TNFα). In fact, it suppresses NF-κB-dependent gene expression, while NF-κB activation is considered to promote survival in cells, inhibiting transcriptional activation [66]. In fact, cycloprodigiosin hydrochloride leads to apoptosis breast [67], liver and colon cancer cells, acting as a H+/Cl− symporter, inducing cytosolic acidification [68]. In addition, it resulted in being useful against promyelocytic leukemia, inducing cell differentiation or apoptosis through up-regulation of Fas ligand, activation of stress-activated protein kinase and caspase [69]. Inhibition of the NF-κB pathway disturbs the immune system, conferring both immunosuppressant and anti-tumor effects [70]. It is also an immunosuppressant agent because of its action as a selective inhibitor of T cell proliferation, like other members of the prodigiosin family [71]. Cycloprodigiosin also stimulates nitric oxide production during hepatic injury, improving cell status by regulating the expression of NF-κB-dependent genes, such as inducible Nitric Oxide Synthase (iNOS) [72], Considering that immunosuppressive drugs such as corticosteroids and mesalazine can prevent the activation of NF-kappaB, both the suppression of NF-κB and increased NO production have been suggested as an anti-inflammatory strategy in inflammatory bowel disease (IBD), so that administration of cycloprodigiosine may limit chronic inflammation [73]. Since NF-κB is known to be a transcription factor regulating inflammatory response genes and implicated in AR, its inhibition could also indicate anti-inflammatory and anti-arthritic properties possessed by cycloprodigiosin [74].

#### 3.3.2. Hymenialdisine

This is an alkaloid isolated from marine sponges, such as *Acanthella aurantica* and *Stylissa massa *[75] and investigated for its properties against NF-κB activation, and its inhibitory effect on IL-8, IL-2 and TNF-α production. Hymenialdisine inhibits several proteins regulating cellular cycle and functions, such as glycogen synthase kinase-3beta, cyclin-dependent kinases, and casein kinase 1, by competing with ATP for binding to these kinases [76]. In this way, it also inhibits phosphorylation of the protein tau (which is hyperphosphorylated in Alzheimer’s disease) with promising potential against human neurodegenerative diseases [77], and NF-κB activity, probably by inhibiting both protein kinase C and I-kB phosphorylation [78]: molecules able to interfere with factors involved in the modulation of gene expression, such as NF-κB, can also be considered as potential anti-inflammatory agents. Its anti-inflammatory properties have also been reported, achieved through its ability to decrease IL-8 [79] and IL-1beta production [80].

In addition, hymenialdisine was tested on bovine articular cartilage, also evaluating its inhibitory effect on proteoglycan degradation in a dose-dependent manner [81]. A potential inhibitory effect on proteoglycan degradation should be investigated on human cartilage and articular damage, typical of RA.

### 3.4. Marine Macrolides

These are class of highly oxygenated natural products, whose structure is characterized by a macrocyclic lactone. The first marine macrolides were the aplysiatoxins, reported in 1974 as toxic constituents of the sea hare *Stylocheilus longicauda*. At present, more than 200 marine macrolides have been discovered, paying attention to their biological active properties, such as immunomodulation, cytotoxic, anticancer, antiviral, and antifungal [82]. Marine macrolides exert antiproliferative cytotoxic activity with various molecular targets [83], representing a promising potential agent in anticancer research [84]. For example, lobophorins A and B are two bioactives with antibiotic, anticancer and anti-inflammatory properties, even stronger than indomethacin [85], and isolated from marine actinomycetes found in the Caribbean brown alga *Lobophora variegata; *latrunculins A and B are architecturally novel molecules isolated from the Red Sea sponge *Latrunculia magnifica*, which also affect cellular growth through disrupting actin polymerization and microfilament organization with antiproliferative effects [86]; aplyronines, isolated from the sea hare *Aplysia kurodai *[87]; dolastatin 19, recently obtained from the sea hare *D. auricularia *from the Gulf of California, which displayed antiproliferative activity, in particular in breast and colon cancer cells [88]; scytophycins, extracted from the blue and green algae *Scytonema pseudohofmanni *[89], and sphinxolides obtained from the New Caledonian marine sponges *Neosiphonia superstes *[90]: They are also actin-binding natural products, able to inhibit the proliferation of human cancer cell lines [91]. In conclusion, macrolides have promising potential, both in anticancer and in rheumatologic research therapy, in terms of their cytotoxic, immunosuppressant and anti-inflammatory properties.

### 3.5. Peptides

#### 3.5.1. Cyclomarins

Cyclomarins are three cyclic heptapeptides (A, B and C), isolated from the marine bacterium actinomycete, belonging to *Streptomyces* sp., along the Californian coast. 

Marine actinomycetes have been exploited as a source of biologically active secondary metabolites with antibacterial and anticancer properties [92]. 

Some molecules have also been reported to be anti-inflammatory, such as cyclomarins and salinamides [93].

Cyclomarin A, constituted of three common and four unusual aminoacids, showed potent anti-inflammatory and antiproliferative activities in both *in vivo* and *in vitro* assays, managing to inhibit edema and pain similarly to the drug hydrocortisone [94].

This molecule also displayed an ability to kill *Mycobacterium tuberculosis* by targeting its caseinolytic protease, resulting in a promising component of antitubercular drugs [95].

A moderate anti-inflammatory effect has been reported also in cyclomarin C, whose total synthesis was recently experimented and reported [96].

For this reason both cyclomarin A and C, and their derivatives, can develop as a potential naturally occurring anti-inflammatory therapies.

#### 3.5.2. Salinamides

These five peptides (A, B, C, D and E) were isolated, like cyclomarin, from marine actinomicetes, belonging to *Streptomyces* *sp*., isolated from the surface of the jellyfish *Cassiopea xamachana*, found in Florida waters [93]. 

Salinamide A and B are the two major bicyclic metabolites, with potent topical anti-inflammatory activity and moderate antibiotic activity against gram-positive bacteria, and could be used in the treatment of tissue inflammation and some infections [97]. 

Salinamides C, D and E are the minor metabolites, whose structure was established through spectral and chemical techniques: salinamide D has a similar structure but contains a valine residue in place of the isoleucine present in salinamide A; salinamides C and E are represented by monocyclic peptides, which exert a light anti-inflammatory activity [98], being potentially able to combat inflammatory diseases.

#### 3.5.3. Halipeptins

These new four metabolites (A, B, C and D) were isolated from the marine sponge *Haliclona,* found in the waters of Vanuatu. Halipeptins are made up of a peptidic portion, conventional alanine residues and unusual residues, assembled in a 17-membered macrolactone ring [99].

Particular attention has been focused on halipeptin A, because of its potent biological activities. Halipeptin A is a cyclic depsipeptide, whose total synthesis has been successfully carried out [100], together with recent syntheses of halipeptin D and its analogues, in order to take advantage of their biological properties [101].

It was found to possess a strong anti-inflammatory activity, both *in vivo* and *in vitro*, even stronger than the classical anti-inflammatory drugs, naproxene and indomethacin [102], resulting in an ability to inhibit edema in mice [99]: this powerful antiphlogistic action is similar to conventional drugs but without their typical side effects, and could be the basis of a beneficial strategy against inflammatory disorders.

### 3.6. Other Metabolites

#### 3.6.1. Petrocortynes

These new lipidic compounds, polyacetylenic alcohols, isolated from marine sponges *Petrosia* [103], were collected from Keomun Island, along the Korean coast [104]. 

*Petrocortyne A* showed cytotoxic activity against solid tumor cells and anti-inflammatory activity inhibiting macrophages, decreasing TNF-alpha production and the expression of migration cell factors involved in phlogistic infiltration [105]. As a consequence, it blocks cellular inflammatory processes and immune cell migration to inflamed tissue; this interferes with the immunopathology of acute or chronic inflammatory and autoimmune diseases, such as septic shock, or rheumatoid arthritis, or even multiple sclerosis, where the pro-inflammatory cytokine TNF-α is widely involved [106]. In addition, not only anti-inflammatory, but also pro-aggregative effects of petrocortyne A have been investigated *in vitro*, establishing that this molecule induces weak intracellular pro-aggregative signals [105,106,107].

*Petrocortyne D*, *E*, *F*, *G *and *H*, whose structures were determined through chemical and spectral methods, exhibited moderate cytotoxicity and inhibitory activity against the enzyme phospholipase A2 [104].

#### 3.6.2. Scytonemin

This alkaloid has an unusual dimeric structure, firstly elucidated in 1993 [108], which can be considered unique among natural products [109].

It was isolated from *Cyanobacteria*, is a yellow to brown, lipid-soluble pigment [110], extracted from the terrestrial alga, Nostoc commune vauch [111]. *Scytonemin* seems to be an inhibitor of polo-like kinase 1 (an enzyme implicated in G2/M transition during the cell cycle) and of platelet-derived growth factor-induced rheumatoid synovial fibroblast. 

In addition, T cells treated with scytonemin were induced to apoptosis, so that it could be developed and used for the treatment of hyperproliferative disorders [109]. Besides this antiproliferative and anti-inflammatory activity, Scytonemin also showed considerable antioxidant activity, so that it could be an interesting therapeutic agent [111] against degenerative anti-inflammatory diseases.

## 4. Antioxidant Agents

Current dietary guidelines in chronic diseases prevention, including cancer and CHD, recommend an increased intake of fruit and vegetables, which are rich sources of antioxidants [112] and have a wide range of antiatherogenic properties [113,114,115]. 

Antioxidants, carotenoids in particular, protect cellular components against oxidative damage, but they have even a role in regulating gene expression and in inducing cell-to-cell communications [116]. Carotenoids are ubiquitously present in nature, existing in plants, algae and microorganisms; they are able to bind heavy metals and toxic substances, such as arsenic, preventing their accumulation in human organisms [117,118]. However, humans and other animals are not able to manufacture carotenoids and require them as part of their diets. There are two classes of carotenoids: carotenes and xanthophylls [119]. Astaxanthin and fucoxanthin are major marine carotenoids, which show strong antioxidant activity, attributed to quenching single oxygen atoms and scavenging free radicals [120].

### 4.1. Astaxanthin

This is the main carotenoid pigment, related to the other well-known carotenoids, β-carotene, zeaxanthin and lutein, found in algae (the chlorophyte alga, *Haematococcus pluvialis*, seems to accumulate the major quantity of astaxanthin in nature) and aquatic animals, present in many popular seafoods (trout, salmon, shrimp, lobster and fish roe) [121]. Astaxanthin contains two additional oxygenated groups on each ring structure compared with other carotenoids, resulting in more powerful antioxidant activities. Its antioxidant property has been demonstrated in several studies: in some cases, it was shown to possess even stronger free radical scavenging and antioxidant activity than vitamin E and β-carotene [122]. It has several essential biological functions: protection against UV light effects, inflammation, aging and age-related diseases, and the promotion of the immune response in the liver, kidney, heart, eyes and joints. It promotes prostate health, protects membranous phospholipids and other lipids from peroxidation [123], and has also been associated with shifts in inflammation response [124]. Clinical studies have also demonstrated reductions in the cardiovascular risk markers of oxidative stress and inflammation, as well as improved blood status [125,126]. Because of its antioxidant and membrane preservation properties, astaxanthin has a considerable potential in the prevention and treatment of various chronic inflammatory disorders, such as cancers, AR, metabolic syndrome, diabetes, diabetic nephropathy, and gastrointestinal liver and neurodegenerative diseases [127], and could provide benefits not only for the cardiovascular system, but also in other inflammatory disease. Therefore, its daily consumption is a practical and beneficial strategy in human health management [128]. 

### 4.2. Fucoxanthin

Fucoxanthin is brown pigment belonging to the class of xanthophylls, with antioxidant properties [129] under anoxic conditions, whereas other carotenoids have practically no quenching abilities, donating electrons as a part of its free-radical quenching function [130]. 

During normal metabolism, the body produces heat: Fucoxanthin affects many enzymes involved in fat metabolism determining an increase in the release of energy from fat [131], thus an increase of thermogenesis. 

Fucoxanthin is a powerful antioxidant that protects cells from oxidative damage and providing other health benefits: improved cardiovascular health, reduction of inflammation, cholesterol and TG levels, improvements in blood pressure levels, and healthy liver function [132,133,134]. 

Future clinical studies will determine the effectiveness of these marine carotenoids (astaxanthin and fucoxanthin) not only on the vascular structure, but also on cartilage and joint health in at-risk patients or in those with established osteoarthritis.

## 5. Conclusion

The sea is a rich source of useful compounds with new chemical structures and pharmacological effects: significant immunomodulation (against allergy), anti-inflammatory (and as a consequence, antitumor and analgesic), antibacterial and antiviral activities [135]. In particular, brown seaweeds contain at the same time many types of bioactives, such as omega-3 polyunsaturated fatty acids (PUFAs), polyphenols, fucosterol, and carotenoids. Algal polyphenols possess many biological activities, including anti-inflammatory, hepatoprotective, anti-tumor, anti-hypertensive and HIV-1 reverse transcriptase activities, as well as anti-diabetic activity, based on the inhibition of α-glucosidase [136]. New marine bioactives, such as COX inhibitors, marine steroids, molecules interfering with NF-κB, macrolides, peptides and many antioxidant agents, could be helpful in treating chronic inflammatory degenerative conditions. Improvement in antioxidant status, through a greater intake of both terrestrial and marine antioxidants, may prevent the development and help the management of inflammatory diseases: circulating antioxidants are scavengers of FRs and may inhibit oxidative damage and lead to the elimination of inflammation. They can not only alleviate symptoms, by reducing disease-related OS, but also tackle the potential side effects of pharmacological therapy, reducing the risk of complications.

The superiority of marine peptides, marine carotenoids, and marine polyphenols, as compared to analogue terrestrial resources, can be explained because of the simultaneous presence in seaweeds of a wider variety of these substances: Their positive actions are synergistic, and so more powerful, as compared with those from terrestrial origins. 

Marine bioactives could potentially develop as functional food, since their biological activities appear to influence the pathogenesis and the clinical course of several inflammatory diseases [137]. Consequently, research should move towards further study and the development of marine functional foods in the hope that, in the future, their regular introduction into the human diet could lead to a reduction in the incidence and severity of many disorders [13]. Considering the lengthening of life expectancy, our eating habits will be crucial in promoting human health. Further research should go in this direction in order to show new preventive and potential therapeutic strategies against several inflammatory chronic diseases [138].
